# A traffic signal and loop detector dataset of an urban intersection regulated by a fully actuated signal control system

**DOI:** 10.1016/j.dib.2023.109117

**Published:** 2023-04-05

**Authors:** Alexander Genser, Michail A. Makridis, Kaidi Yang, Lukas Abmühl, Monica Menendez, Anastasios Kouvelas

**Affiliations:** a Department of Civil, Institute for Transport Planning and Systems, Environmental and Geomatic Engineering, ETH Zurich, Zurich CH-8093, Switzerland; b Department of Civil and Environmental Engineering, National University of Singapore (NUS), 117576 Singapore; c Division of Engineering, New York University Abu Dhabi, Saadiyat Island, Abu Dhabi 129188 United Arab Emirates

**Keywords:** Intelligent transportation systems, Traffic signals, Loop detectors, Signal control systems, Fully actuated systems

## Abstract

Fully actuated signal controls are becoming increasingly popular in modern urban environments, attempting to reduce congestion locally, synchronize flows, or prioritize specific types of vehicles. This trend is expected to grow as more vehicles are expected to communicate via Vehicle-to-Infrastructure (V2I) communication. The presented dataset contains cleaned observations from a fully actuated signal control system with priority for public transportation. Time series data of traffic signals that regulate vehicle, public transportation, bicycle, and pedestrian traffic flows are available, showing where a traffic signal operates in a red or green phase. Also, loop detector data representing the occupancy at several locations at an urban intersection in Zurich, Switzerland is available. The data of all traffic signals and loop detectors corresponds to January and February 2019 and has a resolution of 1 s.

Recent advances in transportation science show novel approaches for signalized intersections, but most publications assess their methodology on self-collected or simulated data. Therefore, the presented dataset aims at facilitating the development, calibration, and validation of novel methodological developments for modeling, estimation, forecasting, and other tasks in traffic engineering. Furthermore, it can be used as a real-world benchmark dataset for objectively comparing different methodologies.

**Specifications Table**

**Table:** 

Subject	Transportation Management
Specific subject area	Urban traffic signal control systems*.*
Type of data	CSV files of traffic signals and loop detector states of an urban traffic signal control system.
How the data were acquired	Text-based log files from a fully-actuated signal control system in Zurich, Switzerland, are processed to acquire time series data of all traffic lights and loop detectors. The log files are processed and cleaned with a Python implementation utilizing regular expressions [1]. The city of Zurich provided the signal control log files that are not publicly available.
Data format	Preprocessed
Description of data collection	A python-script is implemented to receive the log files of the signal control system as input. Then, the relevant and valid traffic signal and loop detector data (timestamp, device type, device id, and device state) are extracted with regular expressions. Finally, a data structure with time series data for all devices at the intersection is designed with a data resolution of 1 s. The implementation of the data parsing and an illustration of feature computation with a data snippet can be retrieved from https://github.com/TrafficControlDataset/data-preparation-suite
Data source location	Institution: Swiss Federal Institute of Technology (ETH) Zurich City/Region: Zurich Country: Switzerland
Data accessibility	Repository name: ETH Research Collection Data identification number: 10.3929/ethz-b-000556642 Direct URL: https://doi.org/10.3929/ethz-b-000556642
Related research article	[2] A. Genser, L. Ambühl, K. Yang, M. Menendez and A. Kouvelas, “Time-to-Green predictions: A framework to enhance SPaT messages using machine learning,” 2020 IEEE 23rd International Conference on Intelligent Transportation Systems (ITSC), 2020, pp. 1–6, doi:10.1109/ITSC45102.2020.9294548. [3] A. Genser, M. Makridis, K. Yang, L. Ambühl, M. Menendez and A. Kouvelas, “Time-to-Green predictions for fully-actuated signal control systems with supervised learning,” arXiv, doi:10.48550/ARXIV.2208.11344

**Value of the Data**

• Due to the scarcity of high-resolution traffic signal and loop detector data from an actuated traffic signal control, this dataset is significant to support the design of methodologies capturing non-linear spatial-temporal traffic relationships.
• Researchers and data scientists can benefit from the dataset when developing and testing estimation and prediction models, e.g., traffic flow, travel times, or signal phase timings.
• The dataset can also be useful for benchmarking to allow for a comparative analysis of various data-driven methodologies at urban intersections.

## 1 Data Description

The dataset consists of four comma-separated-value (CSV) files with 15 days of consecutive time series data each. The corresponding names are ‘intersection_data_set_jan_01_15.csv’, intersection_data_set_jan_16_30.csv’, intersection_data_set_feb_01_15.csv’, and ‘intersection_data_set_feb_16_28.csv’. Note that the second dataset for February only contains 12 days. In total, two months of data are available for January and February 2019. The data includes the device states for all traffic lights and loop detectors (LD) at an urban intersection. Every data sample (a row entry in the dataset) corresponds to one observation. The provided data resolution is 1 s. Each CSV file provides the same data structure defined as follows:

• Time: Time of the recorded sample containing year, month, day, and timestamp in the format YYYY-MM-DD HH:MM:SS.
• sg1 – sg12: Time series of traffic signals 1–12 with corresponding states 0 or 1.
• d1 – d10: Time series of LDs 1 to 10 with corresponding states 0 or 1.

**Fig. 1:**
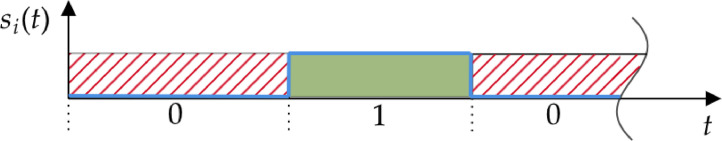
Sample time series of a traffic light i.The signal is highlighted in blue.

Every traffic signal (SG1 to SG12) in the CSV files is represented as a time series with two states. The state 0 indicates that a traffic signal operates in a red phase. State 1 describes a green phase when vehicles/pedestrians are allowed to cross the intersection. Fig. 1 depicts a sample signal of a traffic light (in blue) with two red phases and one green phase (highlighted by the red/green colored areas, respectively. In Switzerland, the beginning and end of a green phase are indicated with a short red/yellow and yellow phase, respectively. As these phases are represented as constants (1 s and 3 s, respectively) and federal legislation does not allow vehicles to cross the intersection, they are assigned to the red phase [4].

**Fig. 2:**
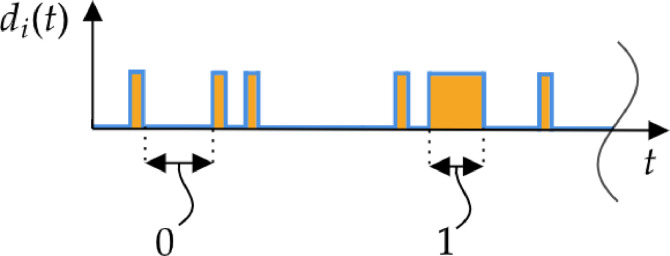
Sample time series of an LD i. The signal is highlighted in blue.

All LDs in the dataset are also provided as time series with the states 0 and 1. The state 0 represents an LD not occupied, i.e., no vehicle currently passes the location where the LD is implemented. On the other hand, if an LD shows a pulse, meaning that the signal state is 1, a vehicle occupies the LD. Fig. 2 depicts a sample signal of an LD with six detections and the corresponding signal states.

### 1.1 Experimental Design, Materials, and Methods

**Fig. 3:**
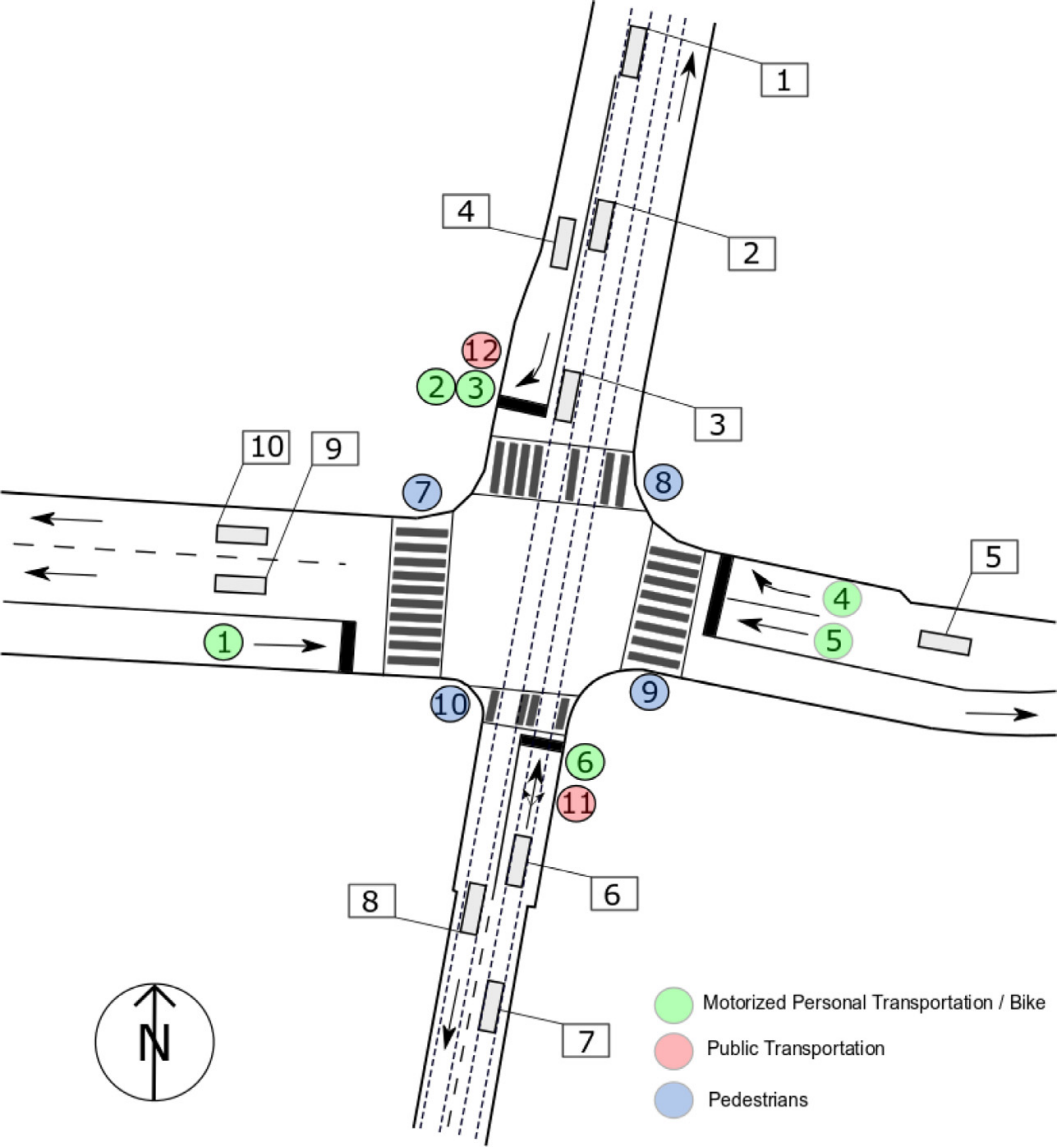
Intersection operated by a fully-actuated signal control system. The circled numbers represent the 12 traffic lights and rectangles with corresponding numbering list available LDs.

The collected dataset represents traffic signal and LD data from a four-leg intersection in Zurich, Switzerland. The intersection, depicted in Fig. 3, is regulated by a fully-actuated signal control system, meaning that red, green, and cycle times are non-constant [5,6]. The variation of these is due to the priority of public transportation and the extension or shortage of signal phases based on the intersection approach's traffic demand [7]. The speed limit for the intersection's northbound and southbound approach is 30 km/h. For approaches from west and east, the maximum speed is regulated at 50 km/h.

In Fig. 3, the traffic signals are shown with circled numbers. There are 12 traffic signals installed at the stop lines. Signals 1, 2, 4, 5, and 6 control vehicular traffic streams and follow a red-green signalization pattern. Traffic signal 3 regulates only bicycle flows that can travel straight to the south. Pedestrian flows are regulated by signals 7–10. From north to south and vice versa, multiple tram lines frequently operate between 7:00 and 22:00. The tram tracks are indicated by dashed lines and overlap with car lanes for the southbound approach. The signal control potentially prioritizes public transportation, and signals 11 and 12 represent the designated traffic lights for trams. This subset of traffic lights only operates in a green phase when trams arrive at the intersection.

The city of Zurich operates the signal control system of the intersection. A centralized system provides log files of the control system that contain event-based telegrams (i.e., records). Every telegram contains the time of the event, an identifier of the control system, the device identifier that triggered the event, and the new device state. For example, if traffic signal 1 changes from a red phase to a green phase, the telegram would contain a device-id of ‘sg1’ and the new state of 1. The log files contain every new event as a one-line string entry. Algorithm 1 contains a pseudo code that allows parsing log files into a tabular dataset. A given list of log files is processed line by line, and the device state information is stored in a new data structure. The implementation is available from https://github.com/TrafficControlDataset/data-preparation-suite. Note that the log details are not included in the implementation as the information is not public.

**Algorithm 1:**
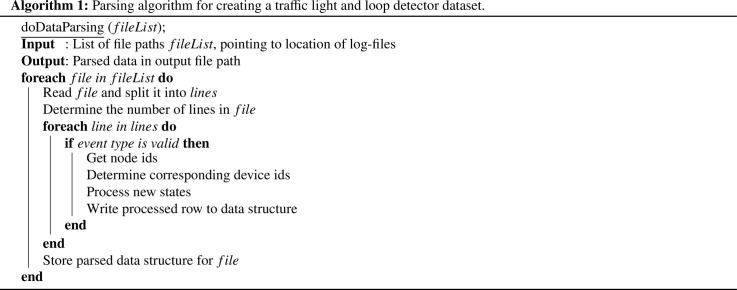
Parsing salgorithm for creating a traffic light and loop detector dataset.

For the compilation of the presented dataset, regular expressions are designed to automatically extract the timestamps, device identifiers, and the corresponding state. The telegrams are event-based, i.e., only when a device changes its state a telegram is sent. Therefore, the time axis is unevenly spaced. We construct a consecutive time axis (resolution of 1 s) and impute the tracked states so that the dataset provides an evenly spaced time series. The procedure is applied for all available log files and all available devices implemented at the intersection.

In the following, details about the location and traffic flows regulated by traffic lights 1–12 are presented. For all available traffic lights, Table 1 represents the location, the controlled transport mode, and the traffic flow characteristics (direction of traffic arrival and departure for all traffic signals, respectively).

**Table 1:** Traffic signals description. MPT=Motorized Personal Transportation; PT=Public Transportation, Sg=Traffic signal.

Device	Location	Transport modes	Direction of arrival	Direction of departure
Sg1	Stop line	MPT, Bike	West	East
Sg2		MPT, Bike	North	West
Sg3		Bike	North	South
Sg4		MPT, Bike	East	West
Sg5		MPT, Bike	East	North
Sg6		MPT, Bike	South	West/North/East
Sg7		Pedestrians	North/South	North/South
Sg8		Pedestrians	West/East	West/East
Sg9		Pedestrians	North/South	North/South
Sg10		Pedestrians	West/East	West/East
Sg11		PT	South	North
Sg12		PT	North	South

For the LDs available in the dataset, Table 2 lists the device name, the location, the detected transport modes, and the traffic light that regulates the detected traffic stream. Note that the location is measured (a) from the stop line if a detector is implemented upstream of a traffic light, and (b) from the pedestrian crossing if implemented downstream.

**Table 2:** Loop detectors description. MPT=Motorized Personal Transportation; PT=Public Transportation, D=Loop detector.

Device	Location	Detected modes	Traffic light
D1	220 m (upstream)	PT	sg12
D2	50 m (upstream)	PT	Sg12
D3	1 m (upstream)	PT	Sg12
D4	18 m (upstream)	MPT, Bike	Sg2, sg3
D5	43 m (upstream)	MPT, Bike	Sg4, sg5
D6	2 m (upstream)	MPT, PT, Bike	Sg6, sg11
D7	15 m (upstream)	MPT, PT, Bike	Sg6, sg11
D8	50 m (downstream)	MPT, PT, Bike	-
D9	10 m (downstream)	MPT, Bike	-
D10	10 m (downstream)	MPT, Bike	-

No separate detector data is implemented for traffic signals 1, 3, and 7–9. Hence, no information on arriving vehicles, cyclists, or pedestrians is available. Note that the LDs in this dataset do not allow for the identification of the vehicle type. LDs 1–3 are able to provide only PT detections, as the sensors are implemented on a dedicated lane for PT.

## Ethics Statements

The authors declare that this work does not involve the use of human subjects, social media data, or experimentation with animals.

## CRediT authorship contribution statement

**Alexander Genser:** Data curation, Conceptualization, Methodology, Software, Visualization, Writing – original draft. **Michail A. Makridis:** Conceptualization, Methodology, Writing – review & editing, Supervision. **Kaidi Yang:** Methodology, Conceptualization, Writing – review & editing. **Lukas Abmühl:** Conceptualization, Methodology. **Monica Menendez:** Conceptualization, Writing – review & editing, Supervision. **Anastasios Kouvelas:** Supervision, Funding acquisition.

## Declaration of Competing Interest

The authors declare that they have no known competing financial interests or personal relationships that could have appeared to influence the work reported in this paper.

## Data Availability

A traffic signal and loop detector dataset of an urban intersection regulated by a fully actuated signal control system (Original data) (ETH Research Collection).
